# A Decade of Transformation in the Management of Childhood Acute Lymphoblastic Leukemia: From Conventional Chemotherapy to Precision Medicine

**DOI:** 10.3390/pediatric17050108

**Published:** 2025-10-16

**Authors:** Maurizio Aricò, Valentino Conter

**Affiliations:** 1 Pediatrics, Ospedale S. Spirito, Azienda Sanitaria Locale, 65124 Pescara, Italy; 2 Centro Tettamanti, Fondazione IRCCS San Gerardo dei Tintori, 20900 Monza, Italy; valentino.conter@gmail.com

**Keywords:** acute lymphoblastic leukemia, pediatric oncology, immunotherapy, minimal residual disease, precision medicine

## Abstract

Over the past decade, the management of childhood acute lymphoblastic leukemia (ALL) has undergone remarkable advancements. This review explores the transition from conventional chemotherapy protocols to precision medicine approaches, highlighting improvements in diagnostic techniques, therapeutic strategies, and personalized treatments within front-line protocols. Key developments include enhanced minimal residual disease detection, the advent of immunotherapies, targeted therapies, and the integration of artificial intelligence. Despite these advancements, challenges remain in ensuring global access and equity. This article discusses the current state of ALL treatment and anticipates future directions in the field.

## 1. Introduction

Acute lymphoblastic leukemia (ALL) is the most common pediatric cancer, representing approximately 25% of all childhood malignancies, with an annual incidence of 3–4 cases per 100,000 children worldwide [1,2]. Over the past decade, remarkable strides have been made in understanding the biology of ALL, leading to improved diagnostic methods and treatment protocols [3,4,5]. These advancements have transformed ALL from a once fatal disease to one with a 5-year overall survival rate exceeding 90% in high-income countries [6,7,8].

The pathophysiology of ALL involves the clonal proliferation of lymphoid precursors with arrested development, more frequently affecting B-cell lineage (85–90% of cases) and less commonly T-cell lineage (10–15% of cases) [9]. The disease is characterized by genetic alterations, including chromosomal translocations, deletions, and mutations that contribute to leukemogenesis [10].

This review aims to provide a comprehensive overview of the major advancements in childhood ALL management over the past decade, highlighting the shift from conventional chemotherapy to more targeted and personalized approaches. We will examine the evolution of diagnostic techniques, therapeutic strategies, and the integration of new technologies that have collectively improved outcomes for children with ALL.

## 2. The State of the Art in 2015: A Foundation for Progress

By 2015, the treatment of childhood ALL had already seen substantial improvements. Standard frontline chemotherapy protocols, such as the Berlin-Frankfurt-Münster (BFM) and Children’s Oncology Group (COG) regimens, were widely implemented, leading to survival rates exceeding 85% in High Income Countries (HIC) [8,11,12]. These protocols typically consisted of induction, consolidation, CNS-directed therapy, delayed intensification, and maintenance phases. Some of the most relevant findings of clinical studies conducted since the 90s are summarized in Table 1. Of note, in recent decades, due to the continuous interaction among pediatric oncology groups, diagnostics, stratification criteria, treatment and indications to hematopoietic stem cell transplantation (HSCT) and outcomes have become progressively similar in HIC.

However, these intensive chemotherapy regimens were associated with relevant short- and long-term toxicities, including infections, organ dysfunction, neurotoxicity, osteonecrosis and secondary malignancies [13,14]. Data from the Childhood Cancer Survivor Study showed that by 30 years post-diagnosis, 73% of ALL survivors developed at least one chronic health condition, and 42% experienced a severe or life-threatening condition [15,16,17,18].

Certain subgroups, such as infants with *MLL* rearrangements and adolescents with high-risk cytogenetic features, continued to have poor prognoses, with 5-year event-free survival (EFS) rates of only 30–40% for infants with *MLL* rearrangements [19,20,21]. Of note, most relapses in these patient subgroups tend to occur early or very early with significantly poorer outcomes [22].

Diagnostic assessments relied on clinical presentation, immunophenotyping, and cytogenetic analyses using techniques like karyotyping, fluorescence in situ hybridization (FISH), and polymerase chain reaction (PCR) [23]. Minimal residual disease (MRD) monitoring emerged as a critical prognostic tool, primarily utilizing flow cytometry (FCM) and real-time quantitative PCR, with detection thresholds of 10^−4^ or above [24]. FCM MRD was shown to be highly prognostic in B-ALL by COG in 2143 patients with MRD response determined in peripheral blood on day 8 and in the bone marrow at the end of induction and at the end of consolidation [25]. In the AIEOP-BFM ALL 2000 study, MRD was assessed in the bone marrow on day 15 by flow cytometry and at the end of induction and of consolidation by PCR, confirming its high prognostic value both in B- and T-ALL [24]. The AIEOP-BFM ALL 2000 trial, including 3184 pediatric patients with B-ALL, demonstrated that patients with MRD levels negative on day 33 had a 5-year event-free survival of 92%, compared to 50% for those with MRD ≥ 5 × 10^−4^ at the end of consolidation; the same study showed that MRD level at the end of consolidation was the strongest prognostic factor in T-ALL [26,27,28]. Similar findings were reported by the COG AALL0232 study, which showed that MRD status at the end of induction was the strongest predictor of outcome [25].

Despite these advancements in frontline treatment, there was a need for more sensitive and standardized MRD detection methods to identify patients at particularly favorable prognosis who could be cured with minimal therapy.

## 3. Transformative Advances in Diagnosis

### 3.1. Enhanced Minimal Residual Disease (MRD) Detection

New methodologies for MRD assessment include:

(1) Digital droplet PCR: Offering improved sensitivity and reproducibility compared to conventional PCR, with a detection limit of approximately 10^−^^5^ [29,30].
(2) Mass cytometry: Allowing for the simultaneous analysis of multiple cellular markers, enhancing the characterization of residual leukemic cells [31].
(3) Next Generation Sequencing MRD: The introduction of next-generation sequencing (NGS) represents the new frontier for MRD detection, enabling the identification of leukemic cells at levels as low as 10^−^^6^ (1 in 1,000,000 cells) [32,33]. This increased sensitivity allows for more precise risk stratification and treatment adjustments, improving patient outcomes. NGS-based MRD assays have demonstrated prognostic significance in both B-cell and T-cell ALL [34].

### 3.2. Advances in Genomic Diagnostics

Recent advances in genomic diagnostics have profoundly improved our ability to characterize the biological heterogeneity of pediatric ALL. High-throughput platforms such as RNA sequencing (RNA-seq) and DNA microarrays enable comprehensive profiling of gene expression patterns, detection of alternative splicing, and identification of cryptic or novel chromosomal rearrangements that are not readily captured by conventional cytogenetics. These tools have become indispensable for uncovering the molecular drivers of leukemogenesis and for refining disease subgroups, thereby supporting risk stratification and treatment tailoring. Importantly, the integration of such approaches into frontline diagnostics has expanded the capacity to monitor minimal residual disease and identify emerging resistance mechanisms.

Among the most clinically relevant discoveries enabled by these technologies are novel fusion genes, such as *MEF2D* and *ZNF384* rearrangements, which define distinct subtypes of B-ALL with characteristic transcriptional profiles, immunophenotypic features, and prognostic implications [35,36,37,38,39]. Patients harboring *MEF2D* fusions often display poor steroid response and inferior outcomes [35,38], whereas *ZNF384* fusions are associated with aberrant expression of myeloid markers and unique therapeutic vulnerabilities [36,37]. Recognition of these molecular subsets underscores the growing importance of precision diagnostics in pediatric ALL, as the identification of such fusions may guide enrollment into molecularly informed clinical trials and open avenues for the development of targeted therapies [38,39,40,41].

## 4. Transformative Advances in Treatment

### 4.1. Hematopoietic Stem Cell Transplantation

Despite the outstanding outcome of front-line chemotherapy, hematopoietic stem cell transplantation (HSCT) remains a critical component of therapy for children with high-risk or relapsed ALL, particularly in cases with poor response to induction therapy, persistent MRD, or high-risk genetic features. The FORUM trial (ALL SCTped 2012 & FOR Omitting Radiation Under Majority age) demonstrated that for children ≥ 4 years old, total body irradiation (TBI) plus etoposide conditioning leads to significantly superior overall survival (OS) and EFS compared to chemotherapy-only myeloablative regimens [42], although long-term health outcomes, particularly regarding CNS toxicity and secondary malignant neoplasm (SMN), should be re-assessed in the coming decades. Long-term toxicities associated with TBI (especially CNS effects, endocrine dysfunction, and secondary malignant neoplasms) underscore the need to optimize conditioning. Advances in donor availability, including the use of haploidentical and cord blood donors, have expanded access to transplantation.

Reduced-Intensity Conditioning (RIC-HSCT): RIC regimens aim to reduce regimen-related toxicity by using less intensive conditioning (lower doses of myeloablative agents or substituting less toxic agents), often in patients who are heavily pretreated, of younger age, or have co-morbidities. Outcomes have been mixed: while RIC can reduce non-relapse mortality, there remains concern about higher relapse rates or decreased graft versus leukemia effect, especially in ALL [43].

Haploidentical HSCT: With donor availability being a major limitation, haploidentical HSCT (haplo-HSCT) has become a promising alternative when matched sibling or unrelated donors are unavailable. Recent data in pediatric ALL show that haploidentical transplants (both T-cell-replete with in vivo depletion or manipulated grafts with techniques such as TCR αβ+/CD19+ depletion) yield similar OS, EFS, relapse incidence, and transplant-related mortality compared to matched donor HSCT in certain settings [44].

Radiation-free (Non-TBI) Myeloablative HSCT: There is an active effort to develop non-TBI myeloablative conditioning regimens to avoid the long-term sequelae of radiation. Chemotherapy-based conditioning regimens—often incorporating agents such as busulfan, cyclophosphamide, fludarabine, thiotepa, or treosulfan—have been studied. Some registry-based studies suggest that for certain patient subgroups (e.g., favorable risk, younger age, negative MRD pre-HSCT), non-TBI regimens may approach outcomes of TBI-based regimens, though generally relapse risk may be higher, particularly for high-risk disease or patients with CNS involvement [45].

Combining CAR-T cells with HSCT may be a promising strategy; however, the benefit of CAR-T-cell therapy bridging allo-HSCT remains controversial [46,47].

Post-transplant relapse remains a challenge, prompting investigations into immune modulation, immunotherapies such as blinatumomab and CAR T-cells, and targeted therapies such as TKIs for Ph+ ALL. Ongoing research is focused on balancing disease control with minimization of late effects, particularly in younger patients, to preserve long-term quality of life.

### 4.2. Immunotherapy Breakthroughs

#### 4.2.1. Blinatumomab

Blinatumomab, a bispecific T-cell engager targeting CD19 and CD3, facilitates T-cell-mediated cytotoxicity against leukemic cells. Common adverse effects include cytokine release syndrome, neurotoxicity, and infections, though these are generally manageable with supportive care.

Blinatumomab was approved, based on the studies mentioned below, by FDA for Philadelphia negative (Ph-) relapsed/refractory B-cell ALL in 2014 for adults and in 2018 for pediatric age. In 2024, blinatumomab was approved by FDA for all patients older than 1 month with Ph- ALL. EMA approved blinatumomab for adults with Ph- relapsed/refractory B-cell ALL in 2015, for MRD-positive ALL in 2019, and as consolidation therapy for all patients in 2025; for pediatric age, blinatumomab is currently approved by EMA only for Ph- relapsed/refractory B-cell ALL. Further approvals may however follow shortly.

The phase III TOWER trial demonstrated that blinatumomab significantly improved overall survival compared to standard chemotherapy in adults with relapsed/refractory B-cell ALL (median 7.7 vs. 4.0 months; hazard ratio 0.71; *p* = 0.01) [48]. In the pediatric setting, the phase I/II RIALTO trial reported a complete remission rate of 47% in heavily pretreated patients [49].

In 2021, COG showed in a randomized study that the substitution of blinatumomab for 2 blocks of chemotherapy for first-high-risk relapse in Ph- pediatric B-cell ALL significantly improved 2-year disease-free survival (from 39.0% to 54.4%; *p* = 0.03) [50]. The benefit of blinatumomab was confirmed by a similar European phase III study which reported a strikingly improved 24 months EFS in patients with similar features randomized for blinatumomab versus chemotherapy (66.2% vs. 27.1% <; *p* ≤ 0.001) [51]. Of note, HSCT was indicated for all patients in these 2 studies. In 2023, COG reported that patients with Ph- first-low-risk B-ALL relapse randomized for the addition of 3 cycles of blinatumomab achieved a significantly better 4-year DFS than those randomized for no-blinatumomab (61.2% ± 5.0% versus 49.5% ± 5.2%; *p* = 0.089) [52].

Last year, COG reported on NEJM a significant improvement of EFS for patients with NCI standard risk features (i.e., age 1–9 years and WBC count < 50,000 at the diagnosis), further classified as average or high risk and randomized to receive 2 non-sequential cycles of blinatumomab in the COG AALL1731 study [53]. These results brought the early closure of the study and the decision by COG to treat with blinatumomab all patients with B lineage-ALL except the very small fraction of NCI standard-risk patients at very favorable prognosis. This strategy is now being progressively implemented in many high-income countries and in some middle-income countries (MIC) too. We remind readers here that the AIEOP-BFM ALL 2017 trial (contemporary to the COG ALL1731 study) randomized patients with B-ALL at medium risk to receive or not one cycle of blinatumomab at the beginning of maintenance, and those at high risk to receive 2 cycles blinatumomab versus 2 intensive chemotherapy blocks; outcome results are still pending. Of note, the WHO has recently included blinatumomab in the Essential Medicines List for children (EMLc).

#### 4.2.2. Inotuzumab Ozogamicin

Inotuzumab ozogamicin, an antibody-drug conjugate targeting CD22, received FDA and EMA approval for relapsed/refractory B-cell ALL in adults in 2017. This agent combines a CD22-targeting antibody with calicheamicin, a potent cytotoxic agent that induces DNA double-strand breaks [54].

The phase III INO-VATE trial demonstrated superior outcomes compared to standard chemotherapy, with complete remission rates of 80.7% versus 29.4% (*p* < 0.001) and median progression-free survival of 5.0 versus 1.8 months in Ph negative relapsed/refractory ALL patients (*p* < 0.001) [48]. In pediatric patients too, preliminary data from ongoing trials have shown promising results, with complete remission rates of 58–67% [55,56].

The main toxicity concern with inotuzumab ozogamicin is hepatotoxicity, particularly sinusoidal obstruction syndrome, which occurs in approximately 11% of patients overall and up to 22% of patients who proceed to HSCT [57,58].

### 4.3. Chimeric Antigen Receptor (CAR) T-Cell Therapy

CAR T-cell therapy involves engineering a patient’s T cells to express chimeric receptors specific to leukemia cells. Chimeric receptors are developed through combining the antigen-binding domain of monoclonal antibodies with intracellular signaling domains of T cell receptors. This approach has led to durable remissions in patients with relapsed or refractory ALL [59,60]. Tisagenlecleucel, a CD19-directed CAR T-cell therapy, received the approval for pediatric and young adult patients with B-cell ALL by FDA in 2017 and by EMA in 2018 based on the results of the ELIANA trial. The ELIANA trial reported an 81% complete remission rate within 3 months of infusion, with 80% of responders remaining MRD-negative in relapsed/refractory pediatric ALL [61]. The 12-month relapse-free survival was 59%, with an overall survival of 76%. Long-term follow-up studies have shown that some patients maintain remission for more than 5 years after a single infusion. Yet, the number of infusions received by those patients was not detailed in the reports. Thus, in the paradigm case of Emily, the child treated at Children Hospital of Philadelphia and remaining in remission at 11 years after single infusion remains a proof-of-principle [62,63,64,65].

Challenges associated with CAR T-cell therapy include several adverse events. Cytokine release syndrome (CRS) occurs in 77–93% of patients, with 27–46% experiencing grade 3–4 toxicity [64,65]. Neurotoxicity is observed in 34–44% of patients, with 10–13% experiencing grade 3–4 events [66]. B-cell aplasia may occur as a result of on-target, off-tumor effects, necessitating immunoglobulin replacement therapy [67]. Antigen loss may be responsible for CD19-negative relapses, occurring in approximately 30% of patients who initially respond to therapy [68].

In addition to Tisagenlecleucel, Inaticabtagene Autoleucel, which is a CD19-directed CAR T-cell approved for relapsed/refractory B-cell ALL in China, is now under trial for children [69].

The risk of T cell malignancies after CAR T- cell therapy may be a concern. A recent report from the DESCAR-T registry database, encompassing all pediatric and adult patients with hematologic malignancies who received CAR T cell therapy in France since 1 July 2018, of 3066 patients including 162 with B cell ALL observed for a median follow-up of 17.7 months, found that only one (0.03%) patient developed a T cell malignancy after CAR T-cell infusion received for a primary cutaneous CD30^+^ T cell lymphoproliferative disorder (anaplastic lymphoma kinase-negative), 3 years after receiving tisagenlecleucel therapy for diffuse large B cell lymphoma [70]. In a multicenter retrospective study of 420 children and young adults with B-ALL who received CD19 CAR T-cell therapy, **7** subsequent malignant neoplasms (1.7%) were identified at a median time from CAR T-cell infusion to SMN of 3.2 years (range, 0.6–8.2). Types of SMNs included hematologic malignancies and solid tumors; many patients had exposure to prior therapies known to increase SMN risk. The rate of SMNs after CD19 CAR T-cell therapy in this cohort is low (~1–2%), similar or slightly lower than comparable cohorts treated with other intensive therapies. Lifelong surveillance remains warranted [71].

To address these challenges, next-generation CAR T-cell products are being developed, including:

(1) BiCAR T cells are dual-specific chimeric antigen receptor T cells engineered to recognize two different tumor-associated antigens, thereby enhancing specificity and minimizing antigen escape mechanisms often seen with single-target CARs [72,73].
(2) TRUCKs (T cells Redirected for Universal Cytokine Killing) represent fourth-generation CAR T cells that, upon antigen engagement, are programmed to secrete immunomodulatory cytokines such as IL-12, thereby augmenting T cell activity and reshaping the tumor microenvironment to overcome immunosuppression [74].
(3) Off-the-shelf allogeneic CAR T-cells: Derived from healthy donors to improve accessibility and reduce manufacturing time [75].

### 4.4. Targeted Therapies

**Figure 1:**
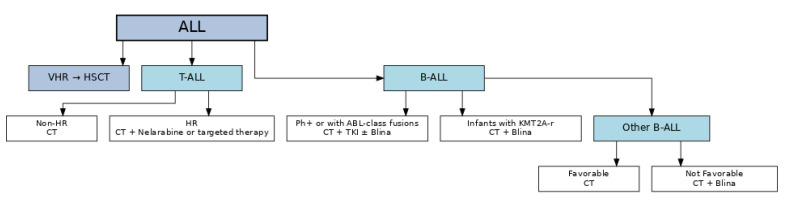
Simplified Algorithm for Pediatric ALL Treatment Strategy.

Advancements in genomic profiling have identified specific genetic mutations and fusions in ALL, paving the way for targeted therapies. Comprehensive genomic analyses have revealed that approximately 80–90% of ALL cases harbor potentially targetable genetic alterations [76,77,78]. The estimated potential use of innovative agents and current approaches in frontline therapy are summarized in Table 2 and in Figure 1, respectively.

### 4.5. Philadelphia Chromosome-Positive ALL

Tyrosine kinase inhibitors (TKIs) like imatinib and dasatinib have transformed the treatment landscape for Philadelphia chromosome-positive (Ph+) ALL, which occurs in approximately 3–5% of pediatric cases [79]. The addition of TKIs to conventional chemotherapy has improved the 5-year EFS from less than 40% to 60% and survival to 80%, with a substantial fraction of patients presenting with relapses (about 30%) [80,81,82,83].

The COG AALL0622 trial demonstrated that replacing imatinib with dasatinib in the COG chemotherapy backbone did not improve the 5-year outcomes [84]. In keeping with these findings, also in the subsequent joint COG-EsPhALL study (CA180-372/COG AALL1122), no improvement of EFS and OS was observed when dasatinib was substituted for imatinib in the EsPhALL chemotherapy backbone, despite decreasing the rate of isolated CNS relapses [85]. A randomized higher dose of dasatinib (80 mg/m^2^ vs. 60 mg/m^2^) compared with imatinib (300 mg/m^2^ vs. 340 mg/m^2^) was associated with improved outcomes in the dasatinib arm with a 4-year EFS of 71% in the Chinese CCCG-ALL-2015 protocol [86]. Yet, extended follow-up is necessary to rule out late relapses and thus document final cure rate in this population. Of note, these results can be obtained while reducing markedly the fraction of patients undergoing HSCT in first remission (to about 10%); HSCT is however generally offered to most patients with Ph+ ALL relapse.

Ponatinib is a recently approved TKI used to treat Ph+ ALL, especially in cases that are resistant or relapsed after other treatments [87].

Nilotinib has also been trialed for pediatric ALL patients. In a phase I study including children (1–<18 y) with Ph+ ALL that were relapsed or refractory, nilotinib (230 mg/m^2^ twice daily) had a safety profile comparable to adults and clinical activity, including complete remissions in some Ph+ ALL patients [88].

### 4.6. Philadelphia-like ALL

Philadelphia-like (Ph-like) ALL, characterized by a gene expression profile similar to Ph+ ALL but without the *BCR-ABL1* fusion, accounts for approximately 15% of pediatric B-cell ALL cases and is associated with poor outcomes [72]. Genetic alterations in Ph-like ALL often activate cytokine receptors and tyrosine kinase signaling pathways, making them potential targets for TKIs [89,90].

Clinical trials incorporating TKIs for patients with *ABL*-class fusions and ruxolitinib for those with *JAK2* alterations have shown promising preliminary results [91]. Thus, the current treatment approach for patients with Ph-like ALL with *ABL*-class fusions is quite similar to the standard therapy for Ph-positive ALL. The COG AALL1521 trial reported that the addition of ruxolitinib to multiagent chemotherapy is feasible and generally well-tolerated; patients often had positive MRD at end of induction before ruxolitinib, with many achieving MRD reduction by end of consolidation [92,93].

### 4.7. Infant Acute Lymphoblastic Leukemia

Infant ALL, defined as leukemia diagnosed before one year of age, represents a biologically distinct and clinically high-risk subset of pediatric ALL. It is characterized by a high prevalence of *KMT2A* (*MLL*) gene rearrangements, which confer poor prognosis and aggressive disease behavior [94,95,96]. Historically, survival rates for infants with ALL have lagged behind older children, with five-year EFS below 50%, as reported in the Interfant-99 study [19]. Recent international collaborative trials, such as Interfant-06, have refined treatment strategies, incorporating risk stratification based on age, white blood cell count, genetic abnormalities, and prednisone response [90]. Emerging data highlight the promise of targeted therapies, such as FLT3 inhibitors and menin inhibitors, particularly in *KMT2A*-rearranged subtypes. However, the most striking results were published in 2023, showing that the addition of a post-induction cycle of blinatumomab allowed a two-year disease-free survival of 81% as compared with 49% in the Interfant-06 trial, with manageable toxicity profiles; the corresponding values for overall survival were 93% versus 66% [97]. Despite these advances, treatment-related mortality remains a significant concern due to the vulnerability of infants to intensive chemotherapy. Ongoing trials are exploring the integration of immunotherapy and precision medicine in current regimens to improve outcomes while minimizing toxicity in this fragile population.

### 4.8. Novel Targeted Agents Under Investigation

The integration of targeted therapies into pediatric ALL protocols is reshaping the treatment paradigm. While the use of TKIs (e.g., imatinib, dasatinib) and immunotherapies (e.g., blinatumomab, inotuzumab, CAR T-cell therapy) has already demonstrated significant benefit in selected subgroups, the therapeutic pipeline continues to expand rapidly.

BCL-2 inhibitors. Venetoclax has shown activity in preclinical models of ALL and is currently being evaluated in combination with chemotherapy in clinical trials [98,99].

FLT3 inhibitors. Agents such as quizartinib are under investigation in *MLL*-rearranged ALL, a subtype often characterized by FLT3 overexpression [100].

Menin inhibitors. These agents disrupt the interaction between menin and MLL fusion proteins. Revumenib, the first-in-class menin inhibitor, received FDA approval in 2024 for relapsed/refractory *KMT2A*-rearranged leukemias, marking a milestone in this high-risk population [101]. Other menin inhibitors, including ziftomenib, enzomenib, and icovamenib, are in early-phase clinical testing and may broaden the therapeutic arsenal.

Epigenetic modulators. Hypomethylating agents and histone deacetylase inhibitors are being studied to address epigenetic dysregulation in ALL [102].

Monoclonal antibodies beyond CD19/CD22. Daratumumab, targeting CD38, is under evaluation in pediatric T-ALL and T-cell lymphoblastic lymphoma, with early reports indicating meaningful MRD responses and acceptable safety. These findings highlight the diversification of immunotherapeutic strategies beyond the established CD19 and CD22 targets.

Taken together, these advances underscore a rapidly evolving field in which translational discoveries are swiftly moving into clinical applications. While most of these therapies remain investigational in frontline pediatric ALL, their successful integration in relapse settings and early-phase trials suggests that personalized, target-directed therapy will become standard for high-risk subgroups in the near future.

## 5. The Role of Artificial Intelligence

### 5.1. Artificial Intelligence in Diagnostics

Artificial intelligence (AI) has been integrated into diagnostic workflows to enhance accuracy and efficiency (Table 3). Tools like FCM-Former, a machine learning-based flow cytometry analysis software, have demonstrated high accuracy in automating the detection of ALL subtypes [103]. These AI-driven approaches assist clinicians in identifying leukemia subtypes, leading to more personalized treatment plans.

Recent advances in artificial intelligence have also facilitated genomic diagnostics in pediatric ALL. Tools such as ALLSorts, an open-source machine learning-based classifier, can accurately assign RNA-seq profiles to defined ALL subtypes, including those characterized by rare fusions like *MEF2D* and *ZNF384*. By automating subtype prediction, ALLSorts and similar AI-driven platforms reduce reliance on specialized bioinformatics expertise for ALL genomics, while providing scalable diagnostic support. This is particularly relevant for resource-constrained settings, where access to advanced molecular pathology is limited, highlighting the potential of AI to democratize precision medicine in childhood ALL [10,104].

### 5.2. Artificial Intelligence in Treatment

Recent applications for AI in ALL management include:

(1) Automated morphological analysis: Convolutional neural networks have achieved >90% accuracy in classifying leukemic cells from peripheral blood smears [105].
(2) Predictive modeling: Machine learning algorithms can predict treatment outcomes and identify patients at high risk of relapse based on clinical, genetic, and MRD data [106].
(3) Drug response prediction: AI models trained on ex vivo drug testing data can predict patient-specific responses to chemotherapeutic agents, potentially guiding treatment selection [107].
(4) Image analysis in flow cytometry: Deep learning algorithms can improve the accuracy and reproducibility of flow cytometry interpretation, reducing inter-observer variability [108].

## 6. Looking Ahead: Promises for the Next Five Years

### 6.1. Personalized Medicine

The integration of genomic profiling into clinical practice enables the development of individualized treatment plans, minimizing toxicities and improving outcomes. By tailoring therapies based on a patient’s genetic makeup, clinicians can select the most effective treatments while avoiding unnecessary side effects [109,110].

Key developments in personalized medicine include:

1. Pharmacogenomic-guided therapy: Adjusting drug dosages based on genetic variants affecting drug metabolism, such as TPMT and NUDT15 polymorphisms for thiopurines [111].
2. Risk-adapted therapy de-escalation: Identifying ultra-low-risk patients who may benefit from reduced treatment intensity, potentially decreasing long-term toxicities [112].
3. Integrated multi-omics approaches: Combining genomic, transcriptomic, proteomic, and metabolomic data to comprehensively characterize leukemia cells and identify therapeutic vulnerabilities [113].

### 6.2. Global Access and Equity

Efforts to reduce the costs of advanced therapies and improve healthcare infrastructure in low- and middle-income countries (LMICs) are essential to ensure that all children have access to these life-saving treatments. While 5-year survival rates exceed 90% in high-income countries, they remain below 40% in many LMICs [114,115].

Strategies to address global disparities include:

1. Simplified treatment regimens: Adapting protocols to be feasible in resource-limited settings while maintaining efficacy [116,117].
2. Point-of-care diagnostics: Developing affordable and portable diagnostic tools for improved risk stratification [118].
3. International collaborations: Establishing partnerships between centers in high- and low-income countries to share expertise and resources [119,120,121].
4. Generic drug production: Promoting the development of affordable generic versions of novel therapies [122].
5. Innovative payment models: Implementing value-based pricing and outcomes-based reimbursement to improve access to expensive therapies [123,124].
6. Global access initiatives: Implementation of programs designed to provide essential ALL medicines at no cost or very low cost—and, where feasible, through commitments by local health authorities. Examples include: the Max Foundation’s patient-assistance programs (e.g., GIPAP and Max Access Solutions), which have provided imatinib and other TKIs at no cost to eligible patients, including those with Ph+ disease [125]; the WHO–St. Jude Global Platform for Access to Childhood Cancer Medicines, which aims to ensure a continuous and reliable supply of quality-assured childhood cancer medicines to approximately 120,000 children in LMICs during 2022–2027 [126]; and the multi-stakeholder ACT for Children initiative, which has begun supplying pegylated L-asparaginase and other essential pediatric oncology medicines to hospitals in LMICs [127], with partners such as Resonance supporting implementation and data systems [128].

## 7. Challenges and Limitations

Despite the remarkable progress in childhood ALL treatment, several challenges remain:

1. Treatment toxicity: Acute and long-term side effects continue to impact quality of life for survivors, with neurocognitive impairment, cardiovascular disease, and secondary malignancies being major concerns [129].
2. Therapy resistance: Approximately 10–15% of patients still experience relapse, often with chemo-resistant disease [130].
3. Cost and accessibility: Novel therapies like CAR T-cell therapy remain prohibitively expensive, with costs exceeding $400,000 per treatment course, excluding hospitalization and management of complications [131].
4. Biomarker standardization: Despite advances in MRD detection, there is still variability in methodologies, reporting, and interpretation across centers [132].
5. Toxicity management: New immunotherapies are associated with unique toxicity profiles that require specialized expertise to manage effectively [133].

## 8. Conclusions

The past decade has seen remarkable progress in the diagnosis and treatment of childhood ALL. From the refinement of MRD monitoring to the advent of immunotherapies and targeted treatments, the landscape has shifted towards more personalized and effective approaches. The integration of genomic profiling, immunotherapy, and artificial intelligence has revolutionized risk stratification and treatment selection, leading to improved outcomes for most high-risk patients.

As the field continues to evolve, the challenge now lies not only in developing the next breakthroughs but also in ensuring that every child—regardless of geography or socioeconomic status—benefits from the advances already achieved. Collaborative efforts between researchers, clinicians, policymakers, and patient advocates are essential to translate scientific discoveries into clinical practice and to address disparities in access to optimal care.

The next years promise further refinement of personalized medicine approaches, expansion of immunotherapeutic options, and innovative strategies to improve global equity. With continued investment in research and healthcare infrastructure, we can envision a future where all children with ALL have access to curative therapy with minimal long-term sequelae. A recent comprehensive review by Kantarjian et al. (2025) provides further context on these developments [134].

## Figures and Tables

**Table 1:** Key Clinical Trials in Pediatric ALL (Past and Recent).

Trial Name	Enrollment Period	Focus and Population	Key Findings
DFCI 95-01	1996–2000	Intensive asparaginase-based treatment	5-year EFS 81.6%, OS 89.6%
COG 1961	1996–2002	Young adults (16–21 years)	Pediatric-style regimen improved 5-year EFS to ~71.5%; limited benefit from HSCT
Interfant-99	1999–2005	Infants, especially *MLL*-rearranged	Poor prognosis in *MLL*+ cases
AIEOP-BFM ALL 2000	2000–2006	MRD-guided risk stratification	MRD is strongest prognostic factor; 5-year EFS ~92% in MRD-negative at end of induction
COG AALL0031	2002–2006	Imatinib for Ph+ disease	Imatinib plus chemotherapy achieved ~80% 3-year EFS; decreased need for HSCT
COG AALL0232	2004–2011	High-risk B-lineage	MRD at end of induction is strongest predictor of outcome
SJCRH Total XV	2004–2010	Risk-directed therapy; no cranial radiation	5-year EFS 85.6%, OS 93.5%; CNS relapse 2.7% without cranial irradiation
COG AALL1731	2015–2019	Randomized Blinatumomab (2 non sequential courses in NCI standard-risk at average or higher risk of relapse	Blinatumomab significantly improved 3-year DFS (96% vs. 87.9%)
AIEOP-BFM ALL 2017	2017–2024	Randomized additional Blinatumomab (one course) in MR patients; randomized 2 courses of Blinatumomab vs. chemo blocks in HR patients	favorable toxicity profile in HR patients; outcome data not yet published
Interfant-06 + blinatumomab	2018–2021	Infants with *KMT2A*-rearranged CD19+ B lineage disease; post-induction Blinatumomab	Phase 2 pilot (n = 30) vs. historical Interfant-06: 2-year DFS 81.6% vs. 49.4%; OS 93.3% vs. 65.8%; good MRD response; acceptable toxicity

**Table 2:** Targeted Therapies and Their Applicability in Pediatric ALL.

Therapy	Target/Indication	Estimated Eligible Proportion
Imatinib/Dasatinib	Philadelphia chromosome–positive (Ph+)	3–5%
Ruxolitinib	*JAK*-mutant/Ph-like	10–15%
Blinatumomab	CD19+ B-lineage	80–85%
Inotuzumab ozogamicin	CD22+ B-lineage	60–70%
CAR T-cell therapy	CD19+ B-lineage; considered in first CR for refractory patients or those with persistent MRD	1–2% of B-ALL

Note. Other targeted therapies, such as rituximab in CD20+ ALL (excluding mature B-ALL), daratumumab in CD38+ ALL, MEK inhibitors (possibly in combination with BCL-2 inhibitors) in cases with *RAS/RAF* mutations, menin inhibitors in *KMT2A*-rearranged ALL, and CAR T-cell therapy in T-ALL, are not yet commonly applied in frontline pediatric protocols.

**Table 3:** AI Applications in Pediatric ALL Management.

Application Area	AI Tool/Function
Diagnostics	AI-assisted immunophenotyping; automated morphological analysis
Risk Stratification	Predictive modeling integrating genetic features and minimal residual disease (MRD)
Treatment Planning	Drug response and therapy optimization models
Global Health Equity	Remote diagnostic support; AI-based classification for underserved regions
